# Two cases of asymptomatic axillary artery occlusion difficult to diagnose preoperatively: pitfalls and its solution in endovascular therapy when approaching from the upper extremity

**DOI:** 10.1186/s40792-019-0670-1

**Published:** 2019-07-27

**Authors:** Ryosuke Nishie, Naoki Toya, Soichiro Fukushima, Eisaku Ito, Yuri Murakami, Takeyuki Misawa, Takao Ohki

**Affiliations:** 1 Department of Vascular Surgery, Jikei University Kashiwa Hospital, Chiba, Japan; 2 Department of Surgery, Jikei University Kashiwa Hospital, Chiba, Japan; 3 Department of Vascular Surgery, Jikei University School of Medicine, 3-19-18, Nishi-shinbashi, Minato-Ku, Tokyo, 105-0003 Japan

**Keywords:** Axillary artery occlusion (AAO), Endovascular treatment

## Abstract

**Background:**

Approaching from the left brachial artery is an important access route in endovascular therapy for complicated aortic and peripheral artery cases.

Here, we report two cases of a poor access route from the left brachial artery because of asymptomatic axillary artery occlusion (AAO), despite no preoperative upper arm blood pressure laterality, a normal ankle brachial pressure index, and absence of occlusion of the subclavian artery on CT scan.

**Case 1:**

Seventy-six-year-old female. We planned endovascular aneurysm repair (EVAR) for para-renal abdominal aortic aneurysm using the snorkel technique in the renal artery, but we failed to pass through the left subclavian artery when approaching from the left brachial artery because of AAO.

**Case 2:**

Seventy-three-year-old female. We planned zone 2 thoracic endovascular aneurysm repair (TEVAR) for thoracic aortic aneurysm and embolization of the left subclavian artery via the left brachial artery, but we failed to pass through the left subclavian artery because of AAO, and therefore, we simply covered the orifice of the left subclavian artery using a stent graft without embolization.

**Conclusions:**

The presence of an asymptomatic AAO may alter the treatment plan but may be difficult to diagnose preoperatively. In those cases in which a brachial or radial artery access is planned, contrast medium should be injected from the contralateral upper extremity during preoperative enhanced CT since the absence of halation of the ipsilateral subclavian/axillary vein provides improved visualization of the AAO which may lead to a better preoperative strategy including the choice of the side of upper extremity access.

## Objective

Recently, endovascular aortic aneurysm repair has made remarkable progress, and it is becoming increasingly common for the access to originate in the upper extremity for complicated aortic aneurysmal cases.

In such cases, a left brachial artery approach is commonly chosen. Since we often encounter asymptomatic left subclavian artery occlusion (LSAO), it is an important consideration when planning the operations.

Here, we report two cases of a poor access route from the left brachial artery because of asymptomatic axillary artery occlusion (AAO), despite no preoperative upper arm blood pressure laterality, a normal ankle brachial pressure index, and no preoperative occlusion of the subclavian artery on CT scan.

### Case 1 (Fig. 1)

A 76-year-old woman (height 156 cm, weight 47 kg) had previously undergone intestinal resection procedures because of superior mesenteric artery occlusion and carotid artery stenting for bilateral internal carotid artery stenosis. As a result, she had a past medical history of old cerebral infarction.

**Fig. 1:**
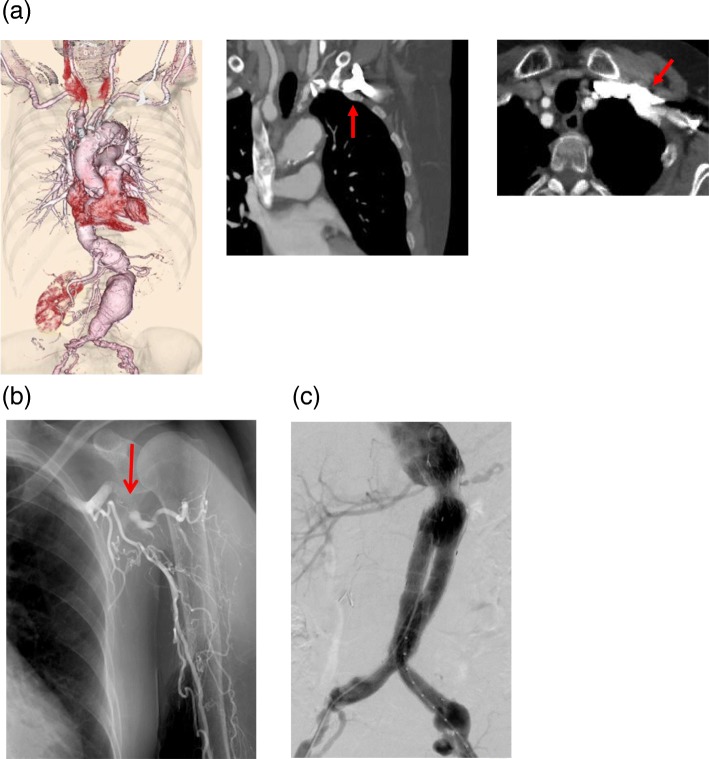
**a** In preoperative enhanced CT scan, AAO was not seen but was well calcified. Contrast is injected from the left upper extremity, and this causes severe halation at the axillar/subclavian vein, which makes visualization of the adjacent left subclavian artery difficult or impossible. **b** AAO was encountered and visualized during operation (arrow). The snorkel technique was abandoned, a regular EVAR was attempted, and accessing the right upper extremity was preserved as a fallback option following failed EVAR. **c** There were no endoleaks on completion angiography despite not performing the snorkel technique

We planned endovascular aneurysm repair (EVAR) for para-renal abdominal aortic aneurysm using the snorkel technique for the renal artery, but we failed to pass through the left subclavian artery when approaching from the left brachial artery because of AAO.

Before the operation, her pulse was palpable and there was no difference between the left and right upper extremity. Her upper arm blood pressure at that time exhibited no laterality (HR 87/min, BP right 149/90 mmHg, BP left 148/84 mmHg).

Preoperative enhanced computed tomography (CT) imaging revealed a para-renal abdominal aortic aneurysm with a maximum diameter of 58 × 63 mm. The axillary artery was densely calcified, but the AAO was not obvious since the contrast medium was injected from the left upper extremity during preoperative enhanced CT, and this caused severe halation at the axillar/subclavian vein, which made visualization of the adjacent left subclavian artery difficult or impossible.

We planned EVAR for her para-renal abdominal aortic aneurysm using the snorkel technique in the renal arteries. We attempted cannulation of the renal artery approaching from the left brachial artery, but failed to pass through the left subclavian artery because of AAO. Therefore, we decided to compromise the EVAR by landing the proximal stent in the short neck without the snorkel technique. Since the completion angiogram showed the absence of endoleak and complete exclusion of the aneurysm, we did not access the right upper extremity to perform the originally planned snorkel technique.

### Case 2 (Fig. 2)

A 73-year-old woman (height 155 cm, weight 49 kg) had right renal arterial embolism, chronic kidney disease, and pleurisy in her past history.

**Fig. 2:**
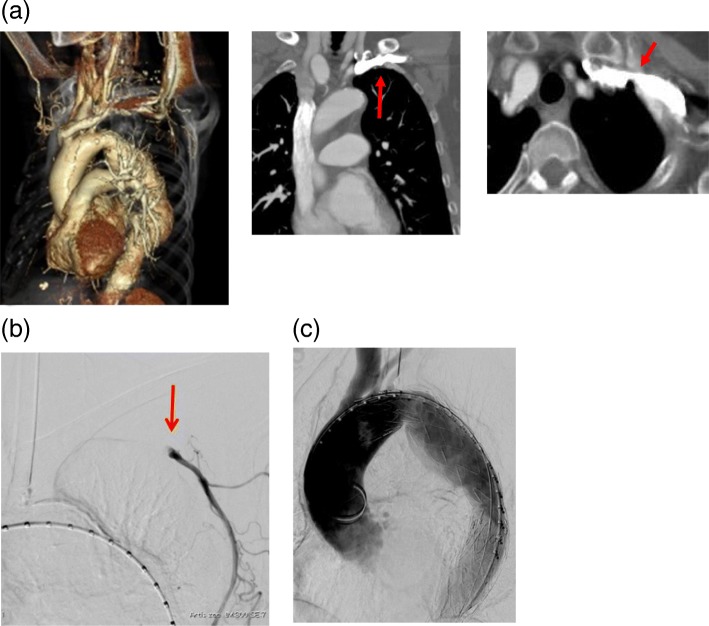
**a** Preoperative enhanced CT scan failed to visualize AAO. (Left innominate vein’s enhancement concealed the presence of AAO.) Contrast was injected from the left upper extremity, and this caused severe halation. **b** The AAO was encountered during LSCA angiography (arrow). **c** There were no endoleaks on completion angiography

Preoperatively, her upper arm blood pressure exhibited no laterality (HR 76/min, BP right 120/80 mmHg, BP left 123/75 mmHg).

Preoperative enhanced computed tomography (CT) imaging revealed a thoracic aortic aneurysm with a maximum diameter of 52 × 50 mm, but the left AAO was not obvious because the left innominate vein’s enhancement concealed LSAO findings.

We planned zone 2 thoracic endovascular aneurysm repair (TEVAR) for thoracic aortic aneurysm and embolization of the left subclavian artery via the left brachial artery, but we failed to pass through the left subclavian artery because of AAO, and therefore, we simply covered the orifice of the left subclavian artery using a stent graft without embolization. No endoleak was observed postoperatively.

## Discussion

The number of surgical strategies has increased due to recent advancement in endovascular treatment. This, in turn, has increased the number of cases requiring access from the brachial artery in the treatment of aneurysms and peripheral artery diseases.

In many of the treatments requiring upper extremity access, approaching from the left subclavian arteries to the aorta and the peripheral arteries is the preferred route because; unlike the right side, it is not necessary to pass across the aortic arch [1–4].

It is well known that peripheral artery diseases or atherosclerosis mostly occurs in the lower extremity, and although less frequent, it does involve the upper arm also. AAO is more prevalent in the left side, and it sometimes makes endovascular treatment difficult when attempting to approach via the left brachial artery [5].

Thoracic outlet syndrome is another cause of AAO and is a well-described disorder caused by thoracic outlet compression of the brachial plexus or the subclavian vessels. Prior reports indicate that subclavian artery compression caused by thoracic outlet syndrome can lead to LSAO, but there have been no reports concerning AAO.

Therefore, the causes of AAO are unclear. Obstruction of the axillary artery in baseball pitchers results primarily from damage to the intima caused by repetitive compression of the pectoralis minor muscle by the tendon, the head of the humerus, or both during pitching [6]. There are some reported cases of arterial injury after shoulder surgery or central venous catheter insertions or radiation therapies causing AAO [7, 8]. In these reports, patients experienced hand numbness, cold sensations, and decreased strength, and radial pulses were not palpable. These cases were diagnosed by enhanced CT scan and angiography [6–8]. However, the 2 cases reported herein had no history of the abovementioned events or causes.

In most cases, including asymptomatic patients, preoperative enhanced CT scan makes it possible to diagnose LSAO, and we can infer its presence by the laterality of upper arm blood pressure. However, it is difficult to detect AAO because preoperative CT scans usually focus on the trunk. Furthermore, injecting contrast material from the veins of the left upper arm makes it difficult to diagnosis AAO because the left innominate vein’s enhancement and halation conceal such findings. We believe that one solution is to inject the contrast from the upper extremity contralateral to the side of anticipated upper extremity access.

In both of our cases, the patients’ radial pulses were palpable and there was no laterality in upper arm blood pressure, so we were unable to anticipate or identify AAO preoperatively.

In our hospital, there have been 150 cases in the past 5 years involving endovascular therapy or angiogram involving access via the left brachial arteries. In two (1.3%) of these cases, AAO findings were observed. In both cases, it was difficult to diagnose AAO preoperatively, but there were similarities in that they were both relatively short, elderly women and experienced mesenteric ischemic events. We believe that the etiology of AAOs in these two cases were caused by atherosclerosis due to the fact that both lesions were densely calcified and also since both had previous medical history of mesenteric ischemia as well as risk factors for atherosclerosis including advanced age, CKD, and stroke.

## Conclusions

The presence of an asymptomatic AAO may alter the treatment plan but may be difficult to diagnose preoperatively. In those cases in which a brachial or radial artery access is planned, contrast medium should be injected from the contralateral upper extremity during preoperative enhanced CT since the absence of halation of the ipsilateral subclavian/axillary vein provides improved visualization of the AAO which may lead to a better preoperative strategy including the choice of the side of upper extremity access.

## Data Availability

Data sharing is not applicable to this article as no datasets were generated or analyzed during the current study.

## Abbreviations

AAO: Axillary artery occlusion
EVAR: Endovascular aneurysm repair
LSAO: Left subclavian artery occlusion
TEVAR: Thoracic endovascular aneurysm repair

## References

[1] Treitl KM, Konig C, Reiser MF, Treitl M (2015). Complications of transbrachial arterial access for peripheral endovascular interventions. J Endovasc Ther.

[2] Millon A, Della Schiava N, Brizzi V, Arsicot M, Boudjelit T, Herail J, Feugier P, Lermusiaux P (2015). The antegrade approach using transbrachial access improves technical success rate of endovascular recanalization of TASC C-D aortoiliac occlusion in case of failed femoral access. Ann Vasc Surg.

[3] Yuan L, Bao J, Zhao Z, Feng X, Lu Q, Jing Z (2014). Transbrachial and femoral artery approach endovascular therapy for flush infrarenal aortic occlusion. Eur J Vasc Endovasc Surg.

[4] Agostoni P, Zuffi A, Faurie B, Tosi P, Samim M, Belkacemi A, Voskuil M, Stella PR, Romagnoli E, Biondi-Zoccai G (2013). Same wrist intervention via the cubital (ulnar) artery in case of radial puncture failure for percutaneous cardiac catheterization or intervention: the multicenter SWITCH registry. Int J Cardiol.

[5] Osiro S, Zurada A, Gielecki J, Shoja MM, Tubbs RS, Loukas M (2012). A review of subclavian steal syndrome with clinical correlation. Med Sci Monit.

[6] Ishitobi K, Moteki K, Nara S, Akiyama Y, Kodera K, Kaneda S (2001). Extra-anatomic bypass graft for management of axillary artery occlusion in pitchers. J Vasc Surg.

[7] Ghanem OM, Sacco J, Heitmiller RF, Gashti SM (2016). Delayed axillary artery occlusion after reverse Total shoulder arthroplasty. Case Rep Orthop.

[8] Aghabiklooei A, Shiva H, Zamani N (2012). Axillary artery occlusion following central venous catheterisation. Emerg Med Australas.

